# Catheter-associated bacterial flora in patients with benign prostatic hyperplasia: shift in antimicrobial susceptibility pattern

**DOI:** 10.1186/s12879-018-3507-9

**Published:** 2018-11-20

**Authors:** Bartosz A. Dybowski, Piotr Zapała, Ewa Bres-Niewada, Łukasz Zapała, Nina Miązek-Zapała, Sławomir Poletajew, Grażyna Młynarczyk, Piotr Radziszewski

**Affiliations:** 1 Department of Urology, Medical University of Warsaw, Lindleya 4, 02-005 Warsaw, Poland; 2 Department of Medical Microbiology, Medical University of Warsaw, Chalubinskiego 5, 02-004 Warsaw, Poland

## Abstract

**Background:**

Men with urinary retention secondary to benign prostatic hyperplasia (BPH) are prone to genitourinary infections. Physicians should be aware of the current antimicrobial susceptibility pattern in this population if empirical treatment is needed. The goal of this study was to evaluate variations in prevalence, composition and antimicrobial susceptibility of bacterial flora in men with indwelling catheters subjected to surgery for BPH in chosen time periods since 1994. Necessary changes in empirical therapy were also assessed.

**Methods:**

All patients with indwelling catheters admitted to a single urological center for BPH surgery in the years 1994–1996, 2004–2006, and 2011–2015 were considered. Catheterization times and results of urine cultures from samples collected at admission were evaluated. Susceptibility for selected antimicrobials was compared separately for Gram negative and Gram positive species. For each agent and for their combinations effectiveness of empirical therapy was calculated dividing the number of patients with bacteriuria susceptible to the agents by the total number of patients with bacteriuria.

**Results:**

Bacteriuria was present in 70% of 169, 72% of 132, and 69% of 156 men in the respective time periods. The incidence of Gram-positive strains increased from 10 to 37% (*P* < 0.001). Their susceptibility to amoxicillin/clavulanate was fluctuating (81, 61, 77%; P=NS). No vancomycin-resistant strain was present. Gram-negative flora composition was stable. Their susceptibility decreased to ciprofloxacin (70 to 53%; *P* = 0.01) and amoxicillin/clavulanate (56 to 37%; *P* < 0.01) while it increased to gentamycin (64 to 88%; *P* < 0.001) and co-trimoxazole (14 to 62%; *P* < 0.001); susceptibility to amikacin remained high (> 85%). Only two cases of resistance to carbapenems in 2004–2006 were found. In vitro effectiveness of amikacin + amoxicillin/clavulanate in empirical therapy was slowly decreasing (87 to 77%; P=NS). Imipenem was found the most effective single agent (90–95%) and its efficacy was even improved by adding vancomycin (97–98%).

**Conclusions:**

Substantial rise in the incidence of Gram-positive species and fluctuations in antimicrobial susceptibility patterns were found. Empirical therapy of genitourinary infection in catheterized men with BPH should now involve antimicrobial agents effective both to Enterococci and Enterobacteriaceae. Periodic monitoring and publishing data on antimicrobial susceptibility for this population is necessary.

## Background

Catheter-associated urinary tract infection (CAUTI) is the most common of all healthcare-associated infections [1]. Despite best care and precautions, long-term catheterization is linked to an almost 100% risk of bacteriuria [2, 3], and up to 80% of catheter-associated strains are resistant to multiple drugs [4, 5]. Permanently or temporarily catheterized patients represent a significant proportion of urological ward patients, and knowledge about resistance patterns is crucial in emergencies when empirical treatment is started. Changes in CAUTI bacterial flora can also be used for monitoring drug resistance in the population because in this patient group, resistant pathogens accumulate as a result of biofilm formation, pressure from antibiotics used for prevention or treatment of CAUTI, and occasional contact with contaminated medical facilities.

Recent European Association of Urology guidelines state that empirical management of CAUTI should be carried out with broad-spectrum antibiotics based on local susceptibility patterns [6]. However, no reports are currently available on the susceptibility of catheterized patients according to the specific reason for catheterization.

Among patients treated at urological departments, the largest group consists of men with urinary retention caused by benign prostatic hyperplasia (BPH) who are admitted for surgical removal of obstruction. In this homogeneous group, we have monitored species composition and resistance patterns since 1994 and have already published on differences after one decade [7]. After another 10-year period, we decided to look again for new trends of resistance in this population. Thus, the primary goal of the current study was to examine the evolution of bacterial species distribution and antibiotic susceptibility patterns of strains isolated from urine of catheterized patients with BPH-related urine retention in a tertiary referral urology department in the last 20 years. The other equally important goal was to consider the necessary changes in the empirical therapy of genitourinary infections in this group of patients. 

## Methods

### Patients and data collection

This retrospective study was conducted at a single academic urological department according to the Declaration of Helsinki. All patients signed informed consent for using their results of routine tests carried out during hospitalization for research purposes. Microbiological data were analyzed for all patients with indwelling transurethral catheters scheduled for surgical treatment of BPH in three time periods: 1994–1996 (Period 1), 2004–2006 (Period 2), and 2011–2015 (Period 3). Patients with other types of obstruction or other methods of urine diversion were not analyzed. In addition, patients without reliable and up-to-date urine culture results were excluded. Data on the presence of bacteriuria, bacterial species, and their resistance to antibiotics as well as time of catheterization measured from installation until hospital admission were obtained from medical records.

### Urine culture collection and analysis

Urine samples for microbiological evaluation were collected during the first 12 h after admission, obtained from catheters during their replacement on ward. Bacterial strains and their resistance were evaluated if significant bacteriuria, defined as > 10^3^ cfu/ml after 24-h incubation at 37 °C, was detected. Contamination was diagnosed when common nonpathogenic strains were grown or more than four strains were found. Bacteria were identified according to standard methods [8]. Isolates were speciated by routine procedures, which included colonial characteristics, Gram-staining, Vitek 2 or Vitek MS (bioMerieux, France) system assays. Susceptibility testing was performed with the disc diffusion method using Mueller Hinton II Agar (MHE, bioMerieux, France) or the VITEK 2 (bioMerieux, France) system. The choice of antimicrobial agents used in susceptibility testing depended on the species of bacteria and the year of examination. Interpretation of test results was based on the recommendations of the Clinical Laboratory Standards Institute to the 2012 and from 2012 to the present in accordance with European Committee on Antimicrobial Susceptibility Testing. Possible susceptibility test results were scored S (sensitive), I (intermediately sensitive), and R (resistant). In further analysis, strains were scored either as sensitive (S and I) or resistant (R).

As a result of differences in antibiotics included routinely in susceptibility testing between the periods of 2004–2006 and 2011–2015, comparative analysis of resistance was based only on drugs used in all analyzed periods and included in the case of Gram-negative bacteria: amoxicillin with clavulanic acid (amox/clav), cefuroxime, cefotaxime, ceftazidime, gentamicin, amikacin, imipenem, ciprofloxacin, cotrimoxazole, and nitrofurantoin. In the case of Gram-positive bacteria amox/clav, imipenem and vancomycin were analyzed. Additionally, for *Enterococci*, the most frequent among Gram-positive isolates, test for the resistance to the high levels of the aminoglycosides (HLAR) was performed using disc diffusion method with discs with gentamycin (120 micrograms) and streptomycin (300 micrograms). In the case of the representatives the Enterobacteriaceae family, test for the detection of the ESBL (Extended-Spectrum Beta-Lactamases) was performed, using discs diffusion method and discs with standard concentrations of aztreonam, ceftazidime, cefepime and amox/clav in the middle. The proportion of susceptible organisms was calculated by dividing the number of urinary isolates resistant to each antibiotic by the number of organisms tested against that antimicrobial agent.

### Statistical analysis

Continuous variables are presented as medians accompanied by ranges or interquartile ranges. Differences among the periods (1994–1996, 2004–2006, and 2011–2015) were evaluated with the Mann–Whitney test for continuous variables and by the chi-square test for categorical variables (including strains and resistance patterns). Influence of catheterization time on bacteriuria occurrence and number of species isolated from a single urine sample was evaluated with Mann–Whitney and rho-Spearman tests, respectively. For all statistical analyses, a two-sided *P* value < 0.05 was considered statistically significant. Statistical analysis was performed using STATISTICA 12 (StatSoft, USA).

## Results

A total of 169, 132, and 156 patients hospitalized in 1994–1996 (Period 1), 2004–2006 (Period 2), and 2011–2015 (Period 3), respectively, met inclusion criteria. Bacteriuria was found in 119 (70%) individuals in Period 1, 95 (72%) in Period 2, and 107 (69%) in Period 3. *Escherichia coli* was the most prevalent uropathogen in all periods (22, 25, 27%), followed by *Proteus mirabilis* (14%) in the 90-ties, and *Enterococcus faecalis* in the Periods 2 (14%) and 3 (19%). All significant species are given in Table 1.

**Table 1:** Bacterial species isolated from urine of men with benign prostatic hyperplasia and indwelling catheters in given time periods

Species	1994–96	2004–06	2011–15	*P* value
*Escherichia coli*	30 (22%)	34 (25%)	40 (27%)	NS
*Enterobacter cloacae*	14 (10%)	11 (8%)	13 (9%)	NS
*Proteus mirabilis*	19 (14%)*	12 (9%)**†**	5 (3%)*†	* 0.002 **† 0.05**
*Klebsiella pneumoniae*	14 (10%)	6 (4%)	14 (9%)	NS
*Klebsiella oxytoca*	1 (1%)	4 (3%)	3 (2%)	NS
*Pseudomonas aureginosa*	17 (12%)*	5 (4%)*	14 (9%)	* 0.01
*Acinetobacter baumannii*	1 (1%)	4 (3%)	3 (2%)	NS
*Citrobacter freundii*	2 (1%)	3 (2%)	5 (3%)	NS
*Serratia marcensens*	1 (1%)	4 (3%)	2 (1%)	NS
Other Gram negative	26 (19%)*	19 (14%)	11 (7%)*	* < 0.01
Total Gram negative	125 (91%)*†	102 (74%)*	110 (73%)†	* < 0.001 † < 0.001
*Enterococcus faecalis*	10 (7%)*	19 (14%)	28 (19%)*	* 0.002
*Enterococcus faecium*	0 (0%)*	8 (6%)*†	2 (1%)**†**	* 0.003 **†** 0.04
*Staphylococcus aureus*	2 (1%)	2 (1%)	1 (1%)	NS
Coagulase-negative staphylococci	1 (1%)	5 (4%)	5 (3%)	NS
Other Gram positive	0 (0%)	2 (1%)	4 (3%)	NS
Total Gram positive	13 (9%)*†	36 (26%)*	40 (27%)†	* < 0.001 † 0.001
Total	138	138	150	

The * or † character was used to determine which pair of values in the row was compared with a statistical test

Median catheterization time was shortest in Period 1 (2 months), followed by Periods 2 and 3 (3 months). For patients with bacteriuria median catheterization time was longer than for those with negative urine cultures in Periods 1 (2 vs 1.75 months; *P* < 0.01) and 2 (3 vs 2 months; *P* < 0.05) but not in Period 3 (3 months in both). Frequently more than one species was isolated from urine of catheterized patients. Two or more species were present in 25 (21%), 37 (39%), and 35 (33%) patients of those with bacteriuria in consecutive Periods. In Periods 2 and 3, catheterization time was positively correlated with the number of isolated strains (rs = 0.3 *P* < 0.01 and rs = 0.25 *P* = 0.02 respectively), but no such correlation was found for Period 1 (rs = 0.18 *P* = 0.06). Gram-positive only bacteriuria was steadily growing. It was found in 10/119 (8%), 11/95 (11.6%), and in 18/107 (17%) patients of those with bacteriuria respectively. Mixed bacteriuria of Gram-negative and Gram-positive organisms was found in 2 (1.7%), 21 (22%) and 21 (19.6%) patients in the consecutive Periods. While all Gram-positive bacteria were susceptible to amox/clav and to imipenem in Period 1, in the following Periods resistant strains of *Enterococcus faecium* and *Staphylococci* were already reported, and susceptibility rate dropped by at least 20%. For Gram-negative species significant changes in both directions in susceptibility to various antimicrobial agents were noted. Detailed susceptibility patterns for Gram-negative and Gram-positive species for all Periods are presented in Table 2. The right choice of empirical therapy can be made with use of data presented in Table 3. It shows in vitro susceptibility patterns of whole bacterial flora identified in samples from individual patients to antibiotics and their combinations. Looking at data in this way amikacin was the most effective choice in Period 1 when susceptibility data for some antibiotics were not available for all patients. In the following Periods efficacy of amikacin alone dropped. In years 2004–06 and 2011–15 its combination with amox/clav was effective in around 80% of patients. At the same time imipenem was the most effective single agent (90–95%). Combination of imipenem with vancomycin increased susceptibility of the flora in specimen to maximum 98%.

**Table 2:** Susceptibility of Gram-negative and Gram-positive strains isolated from patients with indwelling catheters

	1994–96	2004–06	2011–15	*P* value
Gram negative species susceptibility				
No. of isolates	125	102	110	
No. of isolates with reported susceptibility to:				
Amox/clav	ND	56 (56%)	41 (37%)	< 0.01
Cefuroxime	64 (50%)	40 (40%)	52 (47%)	NS
Cefotaxime/Ceftazidime	68 (53%)*	77 (77%) *	84 (76%)	*0.002
Gentamicin	82 (64%)*	75 (75%) *†	97 (88%)†	* < 0.001 † 0.02
Amikacin	120 (93%)	86 (86%)	100 (91%)	NS
Ciprofloxacin	90 (70%) *	53 (53%) *	58 (53%)	*0.01
Cotrimoxazole	18 (14%) *	58 (58%)*	68 (62%)	* < 0.001
Imipenem	ND	98 (98%)	99 (90%)	NS
ESBL +	ND	ND	13 (12%)	–
Gram positive species susceptibility				
No. of isolates	13	36	40	
No. of isolates with reported susceptibility to:				
Amox/clav	13 (100%)	27 (75%)	32 (80%)	NS
Imipenem	13 (100%)	28 (78%)	32 (80%)	NS
Vancomycin	ND	35 (97%)	37 (92%)	NS
HLAR +	ND	ND	12 (30%)	–

Not all agents were tested in all isolates. Only those agents were included that were tested in > 90% isolates. In 2004–2006 resistance to imipenem was found in 2 g-negative strains and in 1 Gram-positive strain. No resistance against vancomycin was found at that period. In 2011–15 resistance to imipenem was found in 2 Gram-negative strains, and resistance to vancomycin to in 1 Gram-postive strain

In years 1994–96 susceptibility to imipenem was not tested, however Gram-positive bacteria susceptible to ampicillin or amoxicillin were considered susceptible to imipenem

The * or † character was used to determine which pair of values in the row was compared with a statistical test

**Table 3:** Susceptibility to selected antibiotics or their combinations of bacterial flora in patients with benign prostatic hyperplasia and indwelling catheters

	1994–96	2004–06	2011–15	*P* value
No. of patients	169	132	156	
No. of patients with bacteriuria	119 (70%)	95 (72%)	107 (69%)	
Bacteriuria susceptible to:				
Ciprofloxacin	76 (64%)*	34 (36%)*	30 (28%)	* < 0.001
Amikacin	102 (86%)*	55 (58%)*	61 (57%)	* < 0.0001
Amox/clav	ND	44 (46%)	44 (41%)	NS
Cefuroxime	51 (43%)*	22 (23%)*	28 (26%)	* < 0.01
Cotrimoxazole	12 (10%)*	27 (28%)*†	49 (46%)†	* < 0.001 † 0.01
Imipenem	ND	86 (90%)	102 (95%)	NS
Amikacin+Amox/clav	103 (87%)	79 (83%)	83 (77%)	NS
Imipenem+Vancomycin	ND	93 (98%)	104 (97%)	NS

Only when all strains isolated from a single patient were susceptible to one antibiotic or to a combination susceptibility in this analysis was recognized. When one or more strains were resistant, intermediate susceptible or not examined for a specific antibiotic non-susceptible status was given. Numbers and percentages of patients with susceptible flora relative to all patients with bacteriuria are reported

*Amox/clav* amoxicillin with clavulanic acid

The * or † character was used to determine which pair of values in the row was compared with a statistical test

## Discussion

This report is the first to specifically characterize bacteriuria in patients with urinary retention secondary to BPH. These patients require special attention of urologists because they are common in our departments, undergo transurethral procedures, that bear high risk of infectious complications and also they may be a source of contamination with multidrug resistance strains. Finally, during several weeks with a catheter, men are prone to infective complications, such as epididymitis, prostatitis, and symptomatic lower urinary tract infections (UTIs). As current urine culture is rarely available when complications arise, empirical therapy is usually implemented and knowledge of antimicrobial susceptibility pattern becomes crucial.

Our data show several characteristic trends in the composition and antimicrobial susceptibility of bacterial flora that occurred during the studied period. First, the percentage of Gram-positive bacteria increased significantly. Whereas in the 90’s it could be assumed that Gram-negative bacteria were responsible for vast majority of CAUTI (90% of patients had Gram-negative species only), later the percentage of patients contaminated with Gram-positive only or with mixed flora involving Gram-positive germs increased significantly. Gram-positive strains in Period 1 were few and all susceptible to amox/clav and imipenem. After ten years appearance of *Enterococcus faecium* and resistant *Staphylococci* caused that those agents had become less effective. At the same time susceptibility Gram-negative flora to fluoroquinolones decreased significantly while susceptibility to cotrimoxazole and gentamicin increased at the same time. The trend for fluoroquinolones has already been reported extensively and attributed to the enormous use of these antibiotics in medicine and in livestock breeding [9]. The improvement in the effectiveness of cotrimoxazole and gentamicin is probably related to lower popularity of both drugs but this issue was investigated to a lesser extent [10, 11]. Whether these trends are universal or not is hard to conclude as results reported by investigators vary.

Studies on antimicrobial resistance of bacteria causing catheter-associated infections are scarce, and none are focused on this specific group of patients. An analysis by Jiménez-Alcaide et al. included all patients with any type of urinary catheter or stent and symptomatic UTI hospitalized in 2012–2013 in a single urological ward [12]. About a quarter of their group consisted of patients with indwelling catheters on admission who might be considered similar to our patients. Another group analyzed samples of urine collected from unspecific catheterized patients in India with UTI symptoms [4]. Valuable data on CAUTI among patients in US acute care hospitals and inpatient rehabilitation facilities also come from reports from the US Centers for Disease Control and Prevention (CDC) National Healthcare Safety Network. A recent update covered the years 2011–2014 [1] and reported on over 138,000 CAUTI events. Data on bacteriuria in catheterized patients in nursing homes from a single Swedish county were presented in a cross-sectional study from 2011 [13], but in contrast to other studies, this group consisted mainly of asymptomatic women and men – only 7% used antibiotics when the sample was collected. However, age, sex, and time of catheterization (70% catheterized longer than 12 months) make this study significantly different from ours. Some CAUTI studies have focused on a specific bacteria species (e.g., Proteus spp. [14]) and others on specific resistance mechanisms (e.g., extended-spectrum beta-lactamase (ESBL) [15, 16]). In those reports, the groups also were not disease-specific, and time of catheterization was not reported.

In contrast, our study reports on a very well-defined group of men with one urological condition, catheterized for a period of several weeks to months. It also offers a 20-year perspective on changes in microbial composition and antimicrobial resistance in a single center, which is unique among papers on CAUTI. Due to the lack of more similar studies, it was decided to compare our results with the aforementioned analyses. A high rate of Gram-positive bacteria in catheter-associated flora was confirmed in some of the mentioned studies (16% [1], 21% [8], 31% [12]) but not all (2% [4]), which may reflect geographical variability. Gram-positive bacteria reported in our series are highly susceptible to amox/clav. However, in the male genitourinary tract, this antibiotic, as well as ampicillin, is not always effective because of low penetration to prostatic tissue [17, 18]. Instead high penetrability has been confirmed for carbapenems and vancomycin [19, 20]. Moreover, *E. faecalis* resistance to amox/clav was already reported to be as high as 20% [12]. With an increasing number of vancomycin-resistant enterococci (VRE) - colonized patients in our department (data on file) and reported by other groups (8% [1]), we predict that BPH patients with catheters colonized by these germs will soon appear.

The choice of antibiotic for treatment of Gram-negative species is a much more difficult task. Rising resistance to fluoroquinolones means that this group, which penetrates very well to prostatic tissue, should not be used any more for empirical treatment of complicated UTIs. A significant decrease in resistance to cotrimoxazole still does not mean that this drug can replace fluoroquinolones. Among agents available in oral formulations, none can be used without urine culture. Despite methodological differences, a high resistance to fluoroquinolones and cotrimoxazole is convincingly confirmed by other studies (17% [13], 33% [1], 50% [12]). The ESBL prevalence of 20% in our group presents the same level as US and Spanish hospitalized patients with CAUTI [1, 12] and among European participants in the SMART study [21]. Toner et al. found that an indwelling catheter almost doubles the risk for ESBL-producing strains in urine [22]. Susceptibility to amikacin among Gram-negative species is still high, but significant prevalence of Enterococci makes this drug poor choice if used alone. Carbapenems is the last resort, however, as in the case of VRE, we can predict growth in the number of carbapenemase-producing strains in the near future. At the Spanish urological department, resistance against amikacin was less frequent than against carbapenems (3% vs 10% for *E. coli*) [12]. The US CDC has reported similar numbers [1]. An older global study on nosocomial UTI showed highest susceptibility to imipenem [23]. A low prevalence of two species, Acinetobacter baumanii and *Klebsiella pneumoniae*, which are the most common producers of carbapenemase, may explain the difference between our results and those in other reports cited here. Apart from geographical variations, exposure to hospital strains also may be a reason. Although our patients visited only outpatient clinics before they entered the ward for surgery, patients from other studies usually were recruited among already hospitalized populations, some of them after urological procedures. In Swedish nursing homes, carbapenemase-bearing strains were also not reported [13]. Considering antimicrobial resistance, our results can be placed between those obtained from hospitalized patients [1, 12] and patients in Swedish decentralized geriatric care facilities where strict adherence to the Swedish Strategic Programme against Antibiotic Resistance recommendations is a rule.

There are some limitations of this study. The main one is the fact that the data come from a single center. Differences in species composition and susceptibility between different continents, countries and centers result from local patterns of antibiotics use, hygienic conditions, migration, or the time of the average catheterization time. Similar analyses should be performed locally in other centers and promotion of this issue was one of our additional goal. Another weakness is the heterogeneous composition of antibiograms. Therefore, only a few antibiotics were repeatedly evaluated in the three Periods studied. Also, we did not have the original minimum inhibitory concentration data and the standard of susceptibility is different prior to and after 2010.

Although our findings do not refer strictly to the situation in other countries and other clinical conditions, we think that taking into account the trends identified in other studies, the significance of these results remains broadly relevant.

## Conclusions

Between 1994 and 2015 substantial rise in the incidence of Gram-positive species in catheter-associated bacterial flora was discovered. This fact, together with fluctuations in antimicrobial susceptibility patterns, make necessary a switch in empirical therapy of CAUTI from Gram-negative targeted agents to therapy effective also to Gram-positive species [24].The choice of drugs in the empirical treatment of genitourinary infection in a male with a catheter was and still is limited to amikacin or carbapenems for *Enterobacteriaceae* and amox/clav, carbapenems or vancomycin for *Enterococci*. The final choice depends on the localization and severity of infection as well as renal function. Monitoring of catheter-associated bacteria resistance patterns reflects trends in the outpatient population, and these surveillance efforts should be continued independent from monitoring hospital-acquired infections.

## Abbreviations

BPH: Benign prostatic hyperplasia
CAUTI: Catheter-associated urinary tract infection
CDC: US Centers for Disease Control and Prevention
ESBL: Extended-Spectrum Beta-Lactamases
HLAR: Resistance to the high levels of the aminoglycosides
UTI: Urinary tract infection
VRE: Vancomycin-Resistant Enterococci
